# Sperm Differentiation: The Role of Trafficking of Proteins

**DOI:** 10.3390/ijms21103702

**Published:** 2020-05-24

**Authors:** Maria E. Teves, Eduardo R. S. Roldan, Diego Krapf, Jerome F. Strauss III, Virali Bhagat, Paulene Sapao

**Affiliations:** 1 Department of Obstetrics and Gynecology, Virginia Commonwealth University, Richmond VA 23298, USA; jerome.strauss@vcuhealth.org; 2 Department of Biodiversity and Evolutionary Biology, Museo Nacional de Ciencias Naturales (CSIC), 28006-Madrid, Spain; 3 Department of Electrical and Computer Engineering, Colorado State University, Fort Collins, CO 80523, USA; diego.krapf@colostate.edu; 4 Department of Physiology and Biophysics, Virginia Commonwealth University, Richmond VA 23298, USA; bhagatvm@mymail.vcu.edu; 5 Department of Chemistry, Virginia Commonwealth University, Richmond VA, 23298, USA; sapaopa@mymail.vcu.edu

**Keywords:** protein trafficking, sperm differentiation, manchette, acrosome

## Abstract

Sperm differentiation encompasses a complex sequence of morphological changes that takes place in the seminiferous epithelium. In this process, haploid round spermatids undergo substantial structural and functional alterations, resulting in highly polarized sperm. Hallmark changes during the differentiation process include the formation of new organelles, chromatin condensation and nuclear shaping, elimination of residual cytoplasm, and assembly of the sperm flagella. To achieve these transformations, spermatids have unique mechanisms for protein trafficking that operate in a coordinated fashion. Microtubules and filaments of actin are the main tracks used to facilitate the transport mechanisms, assisted by motor and non-motor proteins, for delivery of vesicular and non-vesicular cargos to specific sites. This review integrates recent findings regarding the role of protein trafficking in sperm differentiation. Although a complete characterization of the interactome of proteins involved in these temporal and spatial processes is not yet known, we propose a model based on the current literature as a framework for future investigations.

## 1. Introduction

Studies of sperm differentiation in different organisms have revealed how highly organized the process is [1]. Some of the steps in differentiation are shared between species, while others are unique. Generally speaking, sperm differentiation involves the formation of new organelles, chromatin condensation, reshaping of the entire cell by changing nuclear morphology, elimination of residual cytoplasm, and assembly of a flagellum. These events require a cytoskeleton, microtubules, actin filaments and associated motor and non-motor proteins, which effect the efficient delivery of vesicular and non-vesicular cargos to the appropriate intracellular locations. Several trafficking mechanisms have been described. Each of them is spatiotemporally orchestrated, but they differ between species.

This review summarizes recent advances in the field of protein trafficking during mammalian sperm differentiation, highlighting aspects that require further investigation. To that end, we propose a model for the interactome of proteins, emphasizing the need for new approaches for experimental validation. We will present an overview of sperm differentiation from an evolutionary perspective, but attention will be focused on mammalian sperm differentiation. Then, we will examine general aspects of the cellular transformation that takes place during sperm differentiation and, finally, we will concentrate on trafficking of proteins and the interactome that plays an essential role in the differentiation of spermatids to spermatozoa. In order to provide a general perspective, we include an overview of novel technologies to study these processes with a focus on super-resolution imaging. Understanding the trafficking mechanisms and the interactome of proteins may shed light on new approaches for male contraception as well as therapeutic approaches to treat male infertility.

## 2. Evolution of Mammalian Sperm 

Although mammalian sperm share a common structural pattern across species, they differ considerably between taxa, both in shape and size. This begs the question as to how such diversity originates, both with regards to the mechanisms of sperm formation and to the selective pressures that drive the evolution of the male germ cell.

The general structure of the sperm cell consists of a head and a flagellum. The head carries the nucleus with the haploid chromosome set and a secretory granule, the acrosome, that contains enzymes which, upon release in the vicinity of the oocyte, help in penetration of its vestments. The flagellum, which attaches to the head via the neck or connecting piece, consists of three regions, known as the midpiece, the principal piece, and the terminal piece. The midpiece bears the mitochondria that supply energy via oxidative phosphorylation, and the principal piece propels the cell and also contributes to energy generation via glycolysis [2].

Departures from this overall morphological pattern are seen in species that have sperm with no flagellum or that could have two or more. The acrosome may be lacking in some species of fish. In other species, the nucleus may be absent in a subpopulation of spermatozoa that could act as fillers that may carry the nucleus-containing spermatozoa to the site of fertilization. Major differences in sperm shapes make this the cell with the greatest morphological diversity of any cell type [3,4,5]. In mammals, the majority of species have sperm heads that are round or oval, and symmetric but in some taxa (marsupials and rodents), there are major departures from this shape. The sperm head in these taxa could be asymmetric, falciform, with a displacement in the insertion of the flagellum, an acrosome that develops to a large size or to a very small one, and there may be an array of apical or basal appendices that further contribute to asymmetry. These modifications considerably influence the pattern of cell movement and its hydrodynamic efficiency. It is yet not clear how this array of shapes is generated during the period of sperm formation.

The sperm cell is the end result of a highly organized process, known as spermatogenesis, that takes place in the seminiferous tubules of the testis. The process involves three distinct phases. First, a phase of proliferation in which the diploid stem cells, known as spermatogonia, multiply by mitosis. Second, a meiotic phase, in which there is a reduction in the number of chromosomes and that ends with the formation of the haploid round spermatid. Third, the phase in which the cell differentiates from a spheroid to the shape and size typical of each species (spermiogenesis). During this latter period, which may take several days, there are several events including the formation of new organelles such as the acrosome, resulting from the fusion of vesicles originating in the Golgi apparatus, and the chromatoid body, which is composed of RNA. In addition, there is nuclear remodeling and shaping with chromatin condensation and elimination of residual cytoplasm. There is also assembly of the flagellum and relocalization of mitochondria. The process ends with spermiation, the release of the sperm cells into the lumen of the seminiferous tubules.

Spermatogenesis is highly conserved in general terms, although it seems that the first phases are more conserved than the latter ones [6]. The phase of spermiogenesis seems to be more divergent among species, and this may relate to the time required to remodel the sperm nucleus and for the elongation of the flagellum, which appears to be more species specific. The underlying molecular mechanisms may also differ widely, particularly those occurring as part of the communication between the somatic and the germ cells.

The entire process of spermatogenesis occurs with the germ cells receiving support and nourishment from the Sertoli cells (specialized cells that orchestrate germ cell development and differentiation). This seems to be a limiting factor in the production of spermatozoa because the efficiency of cell–cell interactions may limit the number of spermatozoa being supported by Sertoli cells [7]. In fact, the proportion of Sertoli cells in relation to the number of germ cells is directly correlated to the number of spermatozoa produced in the testes and present in epididymal reserves [8].

Diversity in sperm shape and size may be linked to various selective processes. Individuals appear to have the plasticity to adjust the number of sperm produced and, to a certain extent, some morphological features such as sperm size, in response to the perceived risk of potential competition between males. This suggests that males may adjust the processes of sperm formation over a short period of time [9], by modifying the relative size of the testis, its architecture (that is, the proportion of tissue devoted to sperm production) and kinetics [10], and perhaps the molecular processes that underlie sperm differentiation.

At an evolutionary scale, selective forces could favor males that produce spermatozoa with certain shape or size if these traits are important determinants of their reproductive success [11]. When females mate with two or more males, competition between spermatozoa occurs in the female tract, and males with more sperm or sperm cells with faster or more efficient swimming may have advantages in fertilizing oocytes. Females could also exercise some form of selection of one type of sperm over another or impose barriers that sperm need to overcome to reach the site of fertilization. These forces are collectively known as post-copulatory sexual selection [4,11]. An additional powerful selective force is represented by the mode of fertilization. Species with external fertilization release their gametes and, in the rather uniform water environment, sperm need to quickly find the oocyte and engage in fertilization. In species with internal fertilization, the interaction between sperm and the oocyte has become more complex and, in particular, the oocytes are surrounded by thicker layers of cells or coats, and sperm have evolved a series of modified structures to engage in and succeed in fertilization. These modifications include, among others, an enlargement of the enzyme-containing acrosome and a tighter compaction of chromatin [12,13]. One aspect of sperm biology that has probably been influenced by both sperm competition and the mode of fertilization is sperm swimming patterns. Sperm need to swim actively to negotiate barriers in the female tract. Furthermore, they develop a hyperactivated movement after a period of residence in the female tract that allows them to move progressively in the more viscous fluids of the tract. Hyperactivation is important for sperm to generate the propulsive force required to penetrate the zona pellucida. The demands for forceful movement are subserved by both the length of the flagellum and the shape of the sperm head. Sperm with greater size appear to swim faster, and so do spermatozoa with more elongated sperm heads [14,15]. Thus, various selective forces appear to have influenced the design of spermatozoa both in relation to sperm head shape and the size of the different sperm components, particularly the flagellum. This, in turn, may have imposed a need to modify processes of sperm formation, in connection to spatial aspects of seminiferous tubule organization, the kinetics of sperm differentiation and, in addition, the molecular regulation of cellular remodeling.

At the molecular level, the diversity of sperm morphology and function has been influenced by the positive and negative selection of genes. Modifications in amino acid sequences have been selected during evolution [16]. Genes implicated in male reproduction are evolving fast [17], especially proteins essential for spermatogenesis, metabolism, flagellar motility, capacitation, acrosome reaction, and sperm–egg interaction [18]. In particular, genes important for sperm differentiation and protein trafficking appear to be under positive selection. For example, *SPAG17* was shown to be one of the genes that differentiates humans from Neanderthals [19], and its encoded protein has been found to be positively selected [17]. SPAG17 protein was originally characterized as a central pair protein present in the flagellar axoneme [20]. However, this protein is also present in Golgi vesicles, the acrosome and manchette in developing spermatids and has been shown to be essential for sperm differentiation [21]. The *Spag6* gene, another gene originally described to play a role in the central pair of the axoneme [22], has undergone positive selection [23], although there is no change in the amino acid sequence of the protein, and this positive selection seems to be associated with transcriptional control [24]. Recently, a duplication for the *Spag6* gene was found in the mouse, where the *Spag6*-BC061194 gene is the ancestral gene [25]. In spermatids, SPAG6 protein localizes to Golgi vesicles, the acrosome and the manchette. Moreover, *Spag6* knockout results in impaired spermiogenesis [26]. The *IFT88* gene was also selected during evolution [27]. IFT88 protein is present in developing spermatids and localizes to Golgi vesicles, the acrosome–acroplaxome complex as well as the manchette and flagellum [28]. *Ift88^-/-^* mice display impaired spermiogenesis with spermatids carrying several anomalies [29]. Other intraflagellar transport genes were similarly selected during evolution [27] and are essential during spermiogenesis, including *Ift20* [30], *Ift25* [31], *Ift27* [32], *Ift74* [33], *Ift140* [34] and *Ift172* [35].

## 3. General Mechanisms During Sperm Differentiation

### 3.1. Acrosome Development

Acrosome biogenesis in mammals occurs with spermatid differentiation during spermiogenesis. Depending on the species, the number of spermiogenic steps varies (e.g., 16 steps in mice, 17 steps in rats, and 19 steps in humans). Similarly, acrosome biogenesis is divided into a different number of phases. However, in most mammals including humans, acrosome biogenesis can be divided into four major phases.

During the first phase, known as the Golgi phase, that extends from spermiogenesis step 1 to 3 in the mouse, the trans-Golgi system generates numerous proacrosomal vesicles that fuse to form an acrosomal granule in the vicinity of the nucleus. In the cap phase (from steps 4 to 7), the acrosomal granule increases its size and it starts to extend over the nucleus, forming a cap-like structure, gradually covering one-third of the nuclear surface. At this stage, the acroplaxome develops. The acroplaxome anchors the acrosome to the nucleus during spermatid elongation [36]. In the acrosomal phase (from steps 8 to 12), the acrosome migrates to the ventral surface of the elongating spermatid nucleus. At this time, the acrosome condensates and binds to the inner acrosomal membrane. The maturation phase is the last phase and extends from steps 13 to 16. It involves the migration of the acrosome, the acrosomal granule spreads over the entire acrosomal membrane and the acrosome differentiates into anterior and posterior regions. The anterior portion becomes the acrosome apex, while the rest of the acrosome covers a wide area of the nuclear surface, except the part attached to the sperm tail. Acrosome morphology varies from species to species. However, the general structure is divided into an outer acrosomal membrane (OAM) and an inner acrosomal membrane (IAM). The OAM is located immediately below the plasma membrane and the IAM is at a short distance above the nuclear envelope [37].

Several proteins have been shown to be important for acrosome biogenesis. Mutant mouse models involving genes encoding these proteins show defects in the acrosome when vesicle formation and trafficking from the trans-Golgi is impaired, as is the case for mutations disrupting *Pick1*, *Gopc*, *Vps54*, *Hrb*, *Sirt1*, and other genes. Moreover, the defect can be at the level of fusion of vesicles, as is the case for disruption of *Gm130* [38] and AU040320 [39]. There has been a limited number of studies focusing on the interaction and regulation among these proteins. By way of illustration, *Pcsk4*-knockout mice exhibit low expression of ACRBP [40]. ATG7 regulates GOPC [41]. *Gopc*-knockout mice show reduced expression of ZPBP1 and SPACA1. More details about these interactions will be reviewed in the Golgi transport section discussed below.

### 3.2. Nuclear Elongation

**Figure 1:**
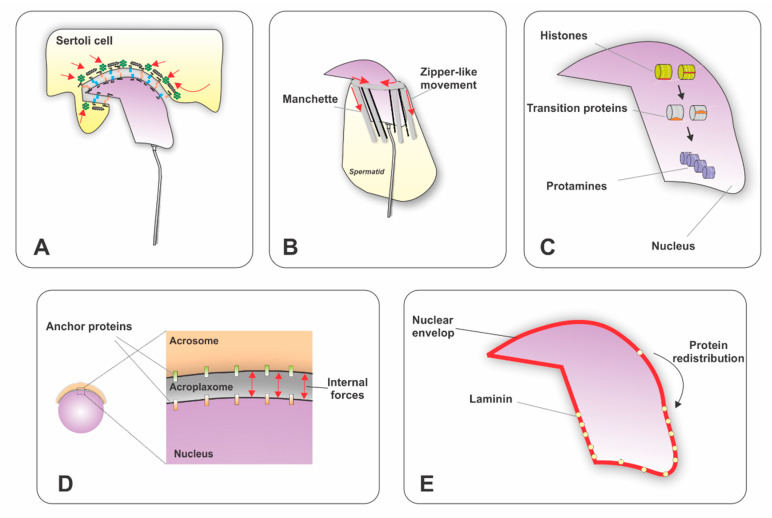
Schematic representation of the processes involved in remodeling of nuclear architecture during sperm differentiation. Illustrations represent mouse spermatids. (**A**) Sertoli cells influence nuclear remodeling by an ectoplasmic specialization that encircles more than one-third of the spermatid head and generates external pressure. (**B**) The manchette, a scaffolding of microtubules and actin filaments, encircles two-thirds of the remaining area of the nucleus. It connects intimately with the nuclear lamina and generates internal forces by a zipper-like movement. (**C**) Replacement of histones by transition proteins and then protamines generates the last twists for chromatin condensation. (**D**) The acroplaxome, which covers more than one-third of the head, modulates the external forces and anchors the acrosome to the nucleus. Additionally, the (**E**) nuclear membrane restructures its molecular composition, redistributing proteins in order to adjust the flexibility of the membrane to allow for external and internal forces. Red arrows indicate the direction of forces.

One of the most remarkable cellular changes accompanying sperm differentiation is the remodeling of the nuclear architecture [42]. In the mouse and rat, in the early steps of spermiogenesis, round spermatids have a spherical nucleus located in the center of the cell and the chromatin is decondensed (steps 1 to 7). The cells then start to form an asymmetric nucleus around steps 9 to 13, and to condense the chromatin around step 12. Several mechanisms have been proposed to influence nuclear elongation and shaping (Figure 1). For instance, (1) Sertoli cells have been shown to modulate nuclear shape through ectoplasmic specializations (ES). At the molecular level, the ES is formed by a layer of actin bundles packed in parallel hexagonal arrays and surrounded by cisternae of endoplasmic reticulum [43]. This structure reduces its diameter and generates external forces to compact the nucleus. (2) The acroplaxome is a cytoskeletal plate composed of F-actin, keratin 5 [36], myosin Va [44] and a marginal ring. Actin-binding proteins localize to this structure and modulate the polymerization/depolymerization of F-actin. For instance, the Arp2/3 complex [45], tyrosine kinases targeting cortactin [46], FerT [47], and the testis-specific proteins profilin-3 (PFN3) and profilin-4 (PFN4) were shown to be crucial for actin microfilament dynamics [48]. It has been proposed that the acroplaxome provides a mechanical planar scaffold modulating external forces generated by the Sertoli cells. It may also play a role in stabilizing and linking the inner acrosomal membrane to the nucleus of elongating spermatids [36]. (3) The nuclear membrane has been shown to influence nuclear shape by changing the distribution of Lamin B3, which generates modifications in the flexibility of the nuclear lamina network [49,50]. Additionally, the LINC complex connects the differentiating spermatid nucleus to the surrounding cytoskeletal structures (microtubule manchette and actin filaments in the acroplaxome) to transfer forces that help with nuclear shaping and elongation [51]. (4) The manchette is a transient perinuclear organelle that is also believed to participate in the elongation of the sperm head and nucleus. It assembles around step 8 during spermatids elongation and disappears when the elongation and condensation of the nucleus is nearly completed (step 14). This organelle consists of bundles of microtubules connected to a perinuclear ring and filaments of actin intercalated between the microtubules [52]. During spermatid elongation, the marginal ring of the acroplaxome and the perinuclear ring of the manchette reduce their diameter as they gradually descend along the nucleus toward the spermatid tail [53] in a zipper-like movement that facilitates nuclear condensation and shaping [54]. (5) The last mechanism proposed to influence nuclear shaping is chromatin condensation via replacement of histones by transition proteins and then protamines [55]. 

### 3.3. Flagellar Formation

In mammals, the assembly of the flagellum starts at early steps during spermiogenesis and continues until sperm release occurs. It initiates with the migration of the pair of centrioles to the cell surface, and then one of the centrioles forms an axoneme (9+2) structure that makes the plasma membrane protrude from the cell. The delicate flagellum rapidly extends into the tubular lumen and the centriole pair forming the axoneme then moves to the nuclear area, and the plasma membrane attached to the centriole folds inward. After contacting the nucleus, the flagellar centriole indents the nucleus to form an implantation fossa. Later, accessory components are added to the flagellum to form the different pieces. Mitochondria are recruited from the cytoplasm to form a helical pattern around the middle piece of the flagellum. Outer dense fibers form both in the midpiece and the principal piece. A fibrous ring is also formed in the principal piece [1].

## 4. Trafficking of Proteins During Sperm Differentiation 

### 4.1. Components of The Delivery System

#### 4.1.1. Tubulin

Several members of the tubulin superfamily have been identified in male germ cells. The α- and β-tubulins are globular proteins with a relative mass of approximately 50 kDa. They assemble to form linear protofilaments, which are the major components of cytoskeletal microtubules. Microtubules are located along the cytoplasm and are one of the main tracks involved in the delivery of proteins. γ-tubulin has been found in the centrosome and basal body and is the key mediator of microtubule assemble and organization. δ-tubulin is present in the perinuclear ring region of the manchette. Other tubulins like ε-, ζ-, and η-tubulin are localized to the basal body and are involved in the centriole and axoneme formation. Tubulins can undergo post-translational modification, such as acetylation, palmitoylation, tyrosine phosphorylation, polyglutamylation and polyglycylation. These modifications play roles in microtubule functions, such as microtubule stability and the interaction with associated proteins. Some of these modifications, such as polyglutamylation, are important for flagellar motility [56].

#### 4.1.2. Actin

Actin is a globular ATPase protein that polymerizes, forming microfilaments. Its relative mass is approximately 42 kDa. The monomeric or globular actin (G-actin) polymerizes, forming helical polymers called F-actin filaments. Based on its isoelectric point, actin is divided into three classes (α, β and γ actin). The amino acid sequences for these actin have been well conserved during evolution [57]. Actin forms a network with associated proteins that participate in the polymerization/depolymerization process as well as in actin binding and regulation (Table 1). Like tubulin, actin can also undergo post-translational modification, such as acetylation, ADP-ribosylation, arginylation, carbonylation, phosphorylation, and methylation [58]. Filaments of actin are essential during sperm differentiation. In spermatids, they are located along the cytoplasm, as well as in the acrosome matrix, acroplaxome, manchette and tail. They participate in nuclear remodeling, acrosome development, and are also tracks for protein trafficking.

#### 4.1.3. Motor proteins

Motor proteins are adenosine triphosphate (ATP)-dependent molecular motors that transport cargo along microtubules and actin filaments, and deliver the contents to specific locations within the cell. The main motor proteins in microtubules are dynein and kinesin. Myosin is the motor protein that drives on tracks of actin filaments.

Several families of kinesin motor proteins have been found in humans and mice, with their molecular structures consisting of three domains: (1) a motor domain that binds to microtubules; (2) a coiled-coil stalk; (3) and the cargo binding region, important for protein–protein interaction [75]. The kinesin molecules and their functions during sperm differentiation are summarized in Table 2. [76].

Dynein consists of a big complex of proteins constituted by two identical heavy chains and a number of associated light chains. The heavy chain contains six AAA domains arranged in a ring. The first four domains have conserved ATP-binding/hydrolysis motifs. Domain AAA1 is essential for dynein motility, while the other sites are believed to play regulatory roles. In contrast to kinesin and myosin, the microtubule-interacting domain is located at the coiled-coil stalk that extends from domains AAA4 and AAA5 [75]. The presence of dynein motors in spermatids has been reported. Cytoplasmic dynein localized to the manchette microtubules [93] and may be important for protein trafficking and nuclear remodeling.

Myosin consists of a diverse superfamily of actin-based motors. There are at least 15 distinct classes of myosin. They have three domains: the head domain, with ATP- and actin-binding sites; the neck domain, interacting with light chains or calmodulin; and the tail domain, responsible for cargo binding. These actin-based myosins have been implicated in diverse functions, such as acrosome biogenesis, organelle and protein transportation during spermiogenesis [57]. Myosin I has a function in post-Golgi vesicle transportation and participates in the biogenesis of the acrosome [94]. In early spermatids, myosin Va binds to Golgi-derived vesicles, is present in the acroplaxome, and is important for intramanchette transport via filaments of actin [36,52,53]. 

#### 4.1.4. Small GTPases (The RAB family)

RAB GTPases are proteins that serve as scaffolds to integrate both membrane trafficking and intracellular signaling in a temporally and spatially sensitive manner. They belong to the Ras superfamily of GTPases. They are small proteins (20–25 kDa), but they have multiple interaction surfaces through which they associate with regulatory molecules and downstream effectors. RAB proteins are present on all compartments of the endomembrane system (endoplasmic reticulum, Golgi, and vesicles), the nucleus, the plasma membrane, mitochondria and centrioles. They regulate anterograde and retrograde protein trafficking between compartments to coordinate cargo delivery to different destinations. RAB GTPases interact with Arf GTPases to enlist proteins present in vesicle coats. Moreover, several RAB proteins (e.g., RAB6, RAB7, RAB11 and RAB27) interact with motor proteins that transport vesicles along microtubules and actin filaments. In addition, RAB proteins also facilitate fusion of membranes by interacting with SNARES [95].

Among the RAB family of proteins, only a few are reported to be expressed in the testis. RAB12 is highly expressed in rat Sertoli cells and plays a role in the transport of vesicles from cellular fringe to perinuclear centrosome region. TBC1D9, a RAB GTPase-accelerating protein (GAP), is abundantly expressed in spermatocytes and is reported to interact with RAB5, RAB7 and RAB9 [96]. RAB3 and RAB27 are present in the acrosomal membrane and are essential for acrosomal exocytosis [97]. RAB27b was detected in the manchette of human and monkey spermatids [98], while RAB10 localizes to the manchette of mouse spermatids [99]. Moreover, RABL2 is essential for sperm tail function and male fertility and RAB8B is known to assist in junction dynamics of the testis [100].

#### 4.1.5. Vesicle Coats

Vesicles develop by budding from membranes and are frequently coated with specific proteins. Three types of vesicle coats have been described. The clathrin-coated vesicles, which are important for endocytosis and vesicular transport from the trans-Golgi to lysosomes, and the non-clathrin-coated vesicles generated from the endoplasmic reticulum (COPII-coated vesicles) and the Golgi (COPI-coated vesicles). The binding of clathrin to membranes is mediated by adaptor proteins. A number of adaptor proteins have been characterized and it is believed that the adaptor proteins select the specific molecules to be transported into the vesicles. Similarly, the coats of COPI- and COPII-coated vesicles form complexes with other proteins that function as the clathrin adaptor proteins. The budding of both clathrin-coated and COPI-coated vesicles from the Golgi complex requires a GTP-binding protein called ARF (ADP-ribosylation factor), while the budding of COPII-coated vesicles from the ER requires a distinct GTP-binding protein called Sar1 [101,102]. Clathrin has been involved in anterograde and retrograde transport of vesicles during acrosome development [103,104,105].

#### 4.1.6. Intraflagellar Transport (IFT) Proteins

Intraflagellar transport is effected by a bidirectional transport system operated by IFT proteins and motors. IFT proteins can be grouped in two network complexes—one named IFTA complex, which includes IFT43, IFT121, IFT122, IFT139, IFT140, and IFT144, and another termed IFTB complex, which includes IFT20, IFT22, IFT25, IFT27, IFT38, IFT46, IFT52, IFT54, IFT56, IFT57, IFT70, IFT74, IFT80, IFT81, IFT88 and IFT172. IFTA interacts with dynein motors and IFTB with kinesin motors to transport proteins along microtubules. IFT transport is essential for mammalian spermiogenesis [29,30,31,32,33,34,35].

### 4.2. Trafficking 

During sperm differentiation, the trafficking of proteins to specific cellular domains plays a key role in the proper development of sperm. Three main trafficking stations distributed in a spatiotemporal fashion have been identified. One of these stations is located between the endoplasmic reticulum, the Golgi apparatus and the acrosome–acroplaxome complex. This will be discussed under Golgi transport. The second station is in the manchette. The relevance of this organelle to sperm differentiation has become evident, and molecular details of its function are now emerging. Moreover, an increasing number of proteins playing a role in this transitory organelle have been reported as well as mutant animal models showing defects in the manchette. We will discuss advances in the knowledge of the trafficking of proteins in the manchette, referred here as intramanchette transport. Assembly of flagellar structures also requires trafficking of proteins. Excellent recent reviews have summarized relevant findings on this subject [106,107,108]. Therefore, we will focus on the first two stations mentioned above. It is worth mentioning, that most of the current knowledge on protein trafficking comes from several studies performed in animal models. However, the mechanisms and the majority of the proteins described below are conserved in humans.

#### 4.2.1. Golgi Transport 

**Figure 2:**
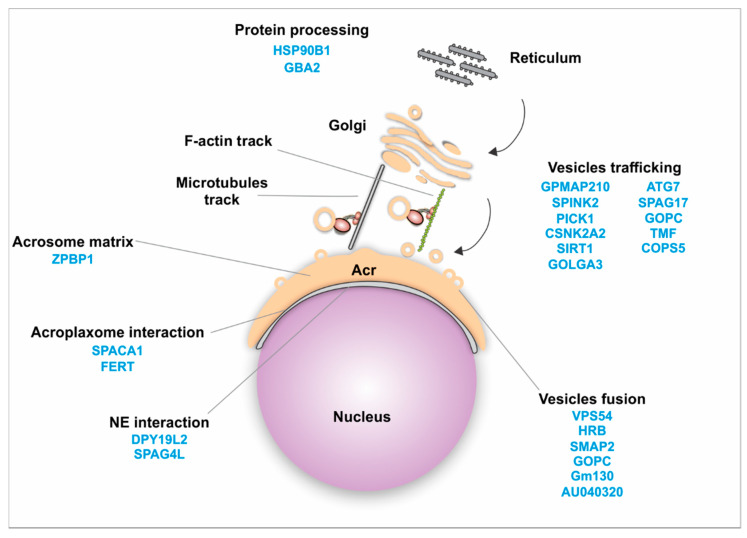
Schematic representation of the Golgi transport mechanism. Several proteins have been identified to play a role in acrosome biogenesis and vesicle transport. Golgi transport involves several steps including protein trafficking from the reticulum to the Golgi apparatus, vesicle transport to the acrosome (Acr) via microtubules and F-actin tracks, vesicle fusion, interaction with the acroplaxome, interaction with the nuclear envelop (NE), and interaction with the acrosomal matrix.

Trafficking of proteins from the Golgi apparatus is essential throughout sperm differentiation. During acrosome biogenesis Golgi-derived vesicles are transported from the trans-Golgi and fused to generate the acrosome. Then they interact with the inner acrosomal membrane, with the acroplaxome, and with the outer and inner nuclear membrane. Microtubules and F-actin tracks present in this region are responsible for this process [52]. Additionally, numerous proteins have been shown to use these tracks for delivery purposes and are important for acrosome biogenesis (Figure 2, Table 3). For instance, the stromal membrane-associated protein 2 (SMAP2) interacts with clathrin assembly lymphoid myeloid leukemia protein (CALM) and syntaxin 2, regulating the assembly of vesicles from the trans-Golgi [109]. The protein interacting with C kinase 1 (PICK1) localizes to the Golgi apparatus in spermatids [110], and is also involved in protein transport. PICK1 regulates the trafficking of vesicles from the Golgi apparatus to the acrosome via interaction with Casein kinase II alpha catalytic subunit (CSNK2A2) [80] and GOPC [111]. GOPC is present in the trans-Golgi region in round spermatids [112]. Golgin subfamily A member 3 (GOLGA3) also interacts with GOPC, contributing to acrosome biogenesis [113,114,115]. TATA element Modulatory Factor (TMF/ARA160) is also required for the fusion of vesicles to the targeted membrane [116,117], and loss of this protein leads to absence of the acrosome, suggesting that TMF/ARA160 influences the transport and docking of proacrosomal vesicles to the nucleus [118]. Autophagy related protein 7 (ATG7) is believed to either facilitate the fusion of vesicles or guide them toward the nucleus [41]. 

Once the Golgi vesicles are transported, they fuse with each other to form the acrosome granule. A number of proteins were reported to facilitate this process. Human Rev-binding (HRB) protein participates in vesicle fusion and also links the Golgi-derived vesicles to the nuclear surface [130]. SMAP2 and GOPC contribute to the fusion of proacrosomal vesicles [109,112]. Vacuolar protein sorting 54 (VPS54) is also important for the fusion of vesicles [145].

Zona pellucida-binding protein 1 (ZPBP1) is another important protein localized to the periphery of the acrosomal membrane [146,167]. Loss of ZPBP1 results in acrosome fragmentation [146]. Attachment of the acrosome to the nucleus is critical for acrosome biogenesis. Sperm acrosome-associated 1 (SPACA1), participates in this process and the disruption of SPACA1 leads to detached acrosome [140]. Fer Testis (FerT) localizes to the cytosolic surface of the outer acrosome membrane and coexists with phosphorylated cortactin in the acroplaxome. These interacting proteins are important for the dynamic regulation of F-actin [47,168].

Subsequently, proteins such as Dpy1912 and SPAG4L assist these proacrosomal granules in attaching to the nucleus [169]. Polypeptide Nacetylgalactosaminyltransferase 3 (GALNT3) is located in the cis-medial region of the Golgi, and its disruption leads to failure of proacrosomal vesicle fusion and transport to nuclear surface [125]. Additionally, developmental pluripotency-associated 19-like 2 (DPY19L2) acts as a bridge between the nucleus and the acroplaxome and its deficiency results in disrupted nuclear/acroplaxome junction, leading to loss of the acrosome [123]. 

Perturbation of the developing acrosome results in male infertility and round-headed spermatozoa (globozoospermia) [53,111,112,130,170]. Other proteins have been shown to localize to the Golgi, Golgi-derived vesicles and the acrosome, including GMAP210 [28,171], IFT88 [28], SPAG6 [26], and SPAG17 [21]. However, their role in acrosome biogenesis has not been elucidated. *Spag17*-knockout mice have deformities in acrosome shape and the acrosome fails to attach to the nucleus [21]. Further studies are needed to understand the mechanism underlying this phenotype. Similarly, conditional deletion of GMAP210 leads to acrosome morphology defects and detached acrosomes [171]. On the other hand, the morphology of the acrosome in the *Ift88* [28] and *Spag6* [26] mutants appears undisturbed.

#### 4.2.2. Intramanchette Transport (IMT)

**Figure 3:**
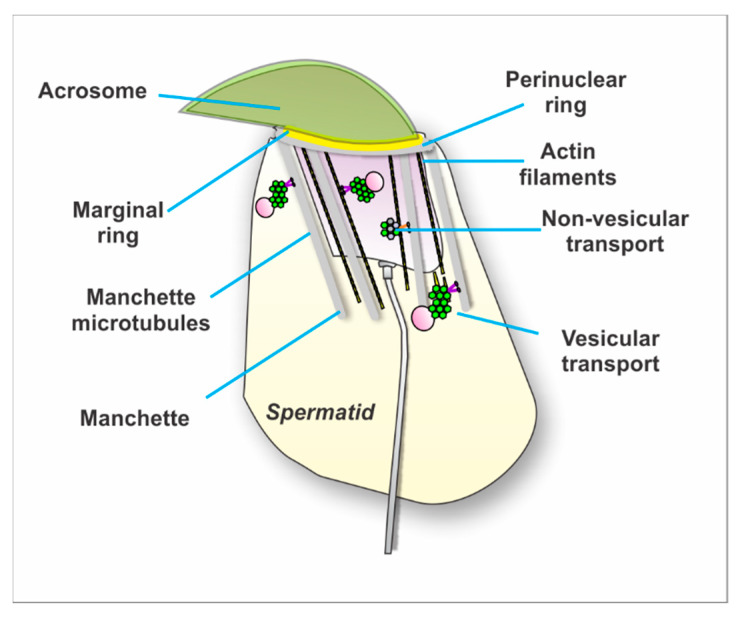
Model illustrating the intramanchette transport mechanism. Illustration represents a mouse spermatid. The manchette is a transitory organelle surrounding the elongating spermatid nucleus. It consists of bundles of microtubules connected to a perinuclear ring and filaments of actin intercalated between the microtubules. Proteins are transported on these tracks to specific intracellular sites during the process of sperm differentiation. Some proteins form large complexes that can transport vesicular as well as non-vesicular cargos.

As mentioned previously, the manchette is a transitory organelle present during spermatid differentiation. It assists with nuclear remodeling but also participates in protein trafficking. This organelle is made up of microtubules and filaments of actin. They provide tracks for cytoplasmic transport of proteins as well as transport between the nucleus and the cytoplasm (Figure 3). In this context, the manchette is tightly bound to the nucleus and proteins are transported through the nuclear pores assisted by intramanchette transport. 

RAN GTPases are key in nucleocytoplasmic transport. RAN exists as RANGTP inside the nucleus and as RANGDP in the cytoplasm. This asymmetric distribution depends on two main regulatory proteins that control the cycling of RAN between these two states—regulator of chromosome condensation 1 (RCC1) in the nucleus and GTPase-activating protein 1 (RanGAP1) in the cytoplasm. RCC1 binds to nucleosomes through interactions with the core of histones H2A and H2B [172]. It has been proposed that this mechanism could participate in the exchange of histones by transitional proteins and protamines during the chromatin condensation process [173]. Other motor proteins, KIFC1 and the nucleoporin protein NUP62, have been shown to interact and participate in the nuclear-cytoplasmic transport mechanism [54]. RANBP17 is a RAN-binding protein also involved in transport in and out of the nucleus. It interacts with SPEM1 and UBQLN1 [174,175]. The exact role of SPEM1 is unknown, but its interaction with UBQLN1 may associate this complex with the degradation of unwanted proteins via the ubiquitin-proteasome system.

An increasing number of proteins have been found to be anchored or harbored around the manchette (Table 4). They assist with nuclear condensation, spermatid differentiation and tail formation [54]. Mutants of these proteins in murine models display perturbations in the manchette and alterations in the nucleus and head shape. However, it is not clear how the trafficking of proteins is coordinated in order to deliver proteins to specific locations. Moreover, the molecular functions of most of the proteins reported to localize to the manchette are largely uncharacterized, and our current knowledge is far too rudimentary to draft a complete interacting protein network. However, some attempts were made to describe these complexes. For instance, PACRG interacts with the meiosis expressed gene 1 (MEIG1). MORN3 [136] and SPAG16L failed to localize to the manchette in spermatids from MEIG1-knockout mice, suggesting that these proteins are dependent on MEIG1 for their transport. RIM-BP3 interacts with HOOK1 and KIF3B [139]. KIF3A directly interacts with MNS1 in the manchette, but MNS1 still localizes to the manchette in KIF3A knockout spermatids [85], suggesting that the transport of MNS1 is not dependent on KIF3A. SPAG17 interacts with SPAG16 and the localization of SPAG16 in the manchette is disrupted in SPAG17 knockout. Interestingly, PCDP1 and IFT20 also fail to localize to the manchette in the SPAG17-knockout mice [21]. However, it is not clear yet whether SPAG17 binds directly to PCDP1 and IFT20 or another linker protein is involved in this complex. Additionally, SPERM1 transports UBQLN1 [174] and RANBP17 [175] in the manchette. The coiled-coil domain containing 42 (CCDC42) protein has been found to be enriched in the perinuclear ring and to colocalize with acetylated-tubulin in the manchette [176]. *Ccdc42*-mutant mice display defects in the manchette [177]. The molecular function of CCDC42 is unknown, but it was shown to interact with ODF1 and ODF2 [176].

The manchette displays microtubule heterogeneity characterized by post-translational modifications of tubulin. These modifications include acetylation and detyrosination of α-tubulin and glutamylation of both α and β tubulin [212]. However, the current map of the manchette heterogeneity needs further evaluation. Additionally, there are several questions that deserve further investigation. For instance, is the sorting of proteins to specific intracellular sites dependent on tubulin and actin post-translational modifications? Is the delivery of proteins exclusive to one mode of transport (microtubules or actin filaments) or can proteins switch from one to the other? It has been hypothesized that proteins can switch from microtubules tracks to actin filament tracks [53]. However, this hypothesis has not been experimentally tested. 

### 4.3. Protein Interactomes

Proteomic studies of different model organisms have provided valuable information relevant to our understanding of the functions of the germ cells and their key conserved elements. However, to the best of our knowledge, there are no proteomics studies focused on the manchette. Isolation of this transitory organelle is not an easy procedure. Moreover, the amount of purified manchettes might not be sufficient for mass spectrometric analysis, if isolated from one subject. However, the information gained from an experiment like this, if successful, would be extremely valuable. 

In an attempt to understand the interactome playing a role in protein trafficking in the intramanchette transport as well as in Golgi transport, we used the Ingenuity Pathway Analysis (IPA), version: 49932394 (https://www.qiagenbioinformatics.com/products/ingenuitypathway-analysis) [213], software from QIAGEN Inc., for protein interaction analysis. We examined the proteins shown in Table 3 and Table 4. The list of proteins and the interacting partners were obtained from references cited in the table. We focused on partners for which protein–protein binding was validated by IP, yeast two-hybrid screen, tubulin-binding assay, affinity purification columns and/or co-transfection. Additionally, the tool “Path Explorer” in the IPA software was used to generate an interactome. This tool elucidates the shortest path between molecules based on specified criteria. Several filters were employed. The software was only allowed to consider “direct” interactions. “Relationship types” were limited to only “protein–protein interactions.” There are several types of molecules, or “node types,” that IPA can consider when making its connections. However, these molecules were filtered to include only the following, in order to capture only proteins or protein complexes: complex, cytokine, enzyme, G-protein coupled receptor, group, growth factor, ion channel, kinase, ligand-dependent nuclear receptor, peptidase, phosphatase, transmembrane receptor, and transporter. “Species” were limited to human, mouse, rat and uncategorized data. All other criteria were left as the default settings. 

**Figure 4:**
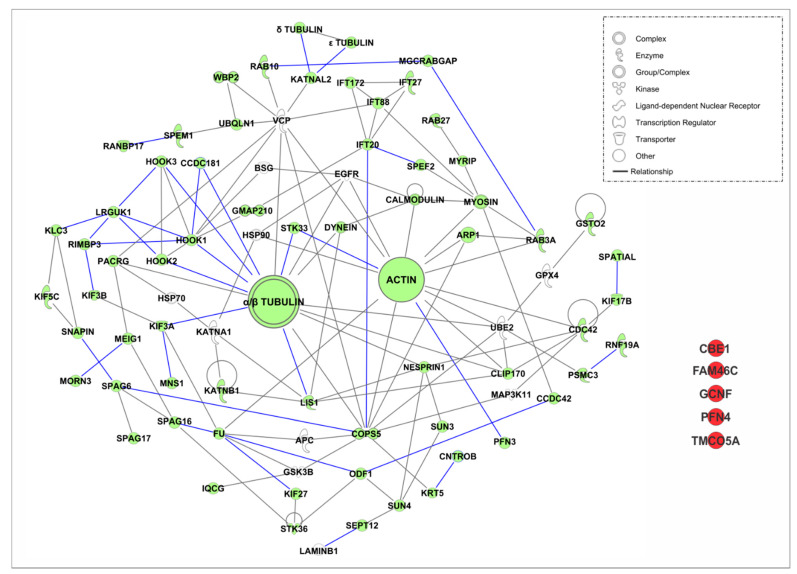
Interactome of proteins involved in intramanchette transport. The Ingenuity Pathway Analysis (IPA), software from QIAGEN Inc., was used for protein interaction analysis. Proteins known to localize in the manchette were analyzed. Only proteins with validated protein–protein binding were included in the analysis. The tool “Path Explorer” in the IPA software was used to generate the interactome. Gray lines are interactions found by the software in IPA databases. Blue lines indicate known interaction validated from current literature by IP, co-IP, yeast two-hybrid screen, tubulin-binding assay, and affinity column purification. Species were limited to human, mouse, and rat.

As expected, due to the number of proteins analyzed, the interactome of proteins shown in Figure 4 to the interactome in the manchette has a complex appearance. All proteins shown in green are proteins from Table 4. Proteins with a white background are new proteins introduced by the software as interacting partners known from IPA databases. Proteins in red are proteins that failed to find an interacting partner in the analysis. We validated the new proteins, and only proteins known to be expressed in male germ cells were included in the figure. Connecting lines in gray are connections made by the software using information from IPA databases. Blue lines are forced connections that we introduced based on literature that confirmed these interactions in the germ cells, as mentioned above. Different shapes are indications for the “type” of protein. Proteins that have a loop connecting back to themselves indicate self-interaction. 

#### 4.3.1. Intramanchette Transport Interactome

The software failed to find interactions for five proteins from our manchette proteins list. This was the case for ciliated bronchial epithelium 1(CBE1), FAM46C, GCNF, testis-expressed profilin 4 (PFN4) and transmembrane and coiled-coil domains 5 (TMCO5A). CBE1 was initially identified in ciliated cells of the bronchial epithelium and it has also been shown to localize to microtubules of the spermatid manchette [181,214]. The role for this protein in the manchette is not clear, but it has been suggested to participate in IMT [181]. Non-canonical RNA polyadenylation polymerase FAM46C is another new protein recently identified in the manchette [185]. Gene knockout of FAM46C in mice results in male sterility, characterized by the production of headless spermatozoa in testes [185]. However, the manchette structures are normal in the mutant mice, suggesting that it may be a cargo protein transported by the IMT. Further studies may be needed to identify its protein partners. GCNF is an orphan member of the nuclear receptor gene family that is essential for embryonic development. In spermatids, this protein localizes to the acrosome, nucleus and manchette [126]. However, the localization in the manchette may be due to its transport in Golgi-derived vesicles to the acrosome or into the nucleus through the nuclear pores [126], suggesting that GCNF may be a cargo protein. Profilins constitute a large and diverse protein family which are crucial for actin microfilament dynamics. Recently, PFN3 and PFN4 were reported to localize to the manchette. However, only PFN3 was confirmed to bind to β-actin and PFN4 seems to lack actin-binding domains in its structure [48]. Therefore, it might not be involved in actin dynamics as is PFN3. Further studies are needed to determine its interacting partners as well as the function this protein plays in the manchette. TMCO5A has been recently detected in the manchette [204]. However, its function in this organelle and the interacting proteins are still unknown. Because of localization in the Golgi, it has been suggested that TMCO5A may be involved in vesicle transport in the manchette [204], but this needs further experimental confirmations. As a whole, the proteins that failed to be incorporated into the interactome deserve further study in order to understand their roles in the manchette and to identify their interacting partners.

Significantly, additional interacting partners were identified by the software. GPX4 is an enzyme required for the structural integrity of sperm chromatin. It has been shown to localize to the nuclear matrix of spermatids as well as in the acrosome and nuclear matrix of sperm [208]. Ubiquitin-conjugating enzyme E2 (UBE2) is a member of the ubiquitin proteasome system. Expression of this protein has been detected in rat spermatogonia [165]. The glycogen synthase kinase (GSK3B) is a serine/threonine protein kinase involved in a large number of cellular processes, including glucose regulation, inflammation, and immune responses, proliferation, migration, and apoptosis [209]. The anaphase promoting complex (APC) is a multisubunit E3 ubiquitin (Ub) ligase that assembles polyubiquitin chains on substrate proteins and targets them for 26S proteasome-mediated degradation [148]. The epidermal growth factor receptor (EGFR) has been shown to be present in the acrosome of boar sperm and plays a role in acrosome reaction signaling [207]. BSG or Basigin is a highly glycosylated transmembrane protein that belongs to the immunoglobulin superfamily. In the testis, BSG is expressed in Sertoli cells, Leydig cells and all stages of germ cells. BSG-knockout mice display impaired spermatogenesis [206]. Heat shock protein 90 (HSP90) and heat shock protein 70 (HSP70) are heat shock family molecular chaperones and both are present in the testis [155,210]. KATNA1 is a microtubule severing protein expressed in multiple tissues, including testis. Loss of this protein results in defects in spermatogenesis [133]. The valosin-containing protein (VCP) is a ubiquitin-binding protein dislocase playing a role in vesicular trafficking and autophagia [166]. The mitogen-activated protein kinase kinase kinase 11 (MAP3K11) localizes to the cytoplasm of late pachytene spermatocytes and round spermatids, in addition to the developing acrosome in spermatids. This protein may be involved in the phosphorylation of target proteins associated with sperm acrosome development [211]. Altogether, it is unknown whether these proteins localize to the manchette. Therefore, it will be important to further investigate these proteins. 

The software was also able to identify proteins that are known to be part of a complex. For example, the PACRG–MEIG1 complex is important for the transport of SPAG16L and MORN3 [136,194]. Interestingly, the software found VCP and HSP70 as new interacting partners for PACRG. Moreover, an IFT complex (IFT20, IFT27, IFT88 and IFT172) is linked to SPEF2 and COPS5 via IFT20. IFT20 may also interact with HOOK proteins using GMAP210 as a linker. Remarkably, CLIP170, VCP, UBE2M, EGFR and GOPS5 are linked to both actin and tubulin, suggesting that proteins can switch from one track to the other. IFT88 and CCDC42 are also linked to actin tracks via myosin. 

Additionally, VCP and COPS5 have several interactions, suggesting that these proteins may play and essential role in the interactome. Consistently, targeted deletion of VCP in mouse results in early embryonic lethality [215]. Moreover, conditional deletion of COPS5 in male germ cells results in impaired spermatogenesis. Mice deficient in COPS5 show dramatically reduced sperm numbers, defects in acrosome biogenesis, absence of acroplaxome and increased apoptosis in germ cells [122]. 

#### 4.3.2. Golgi Transport Interactome

A number of proteins have been shown to participate in Golgi transport. However, as for IMT, the complete interactome of proteins is unknown. Therefore, a similar analysis using IPA software was performed. The list of proteins from Table 3 was analyzed using the same parameters previously indicated.

**Figure 5:**
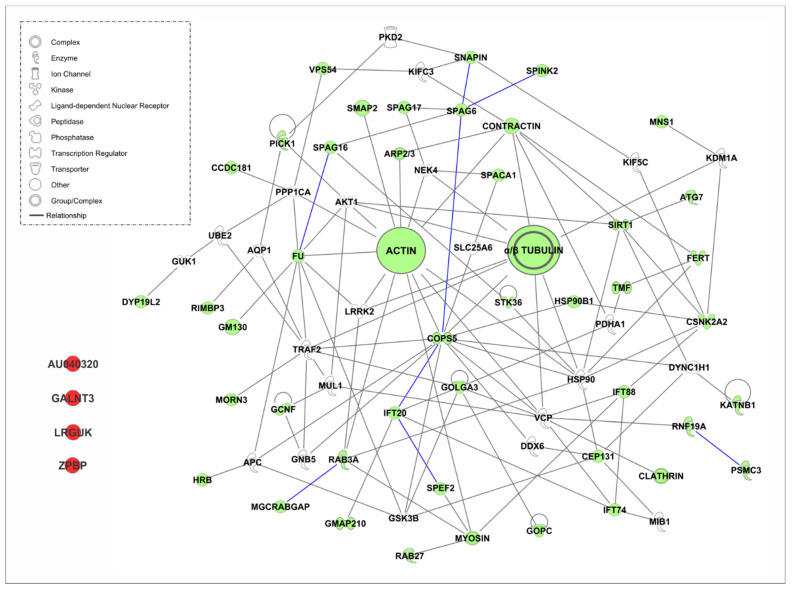
Interactome of proteins involved in Golgi transport. The Ingenuity Pathway Analysis (IPA), software from QIAGEN Inc., was used for protein interaction analysis. Proteins known to participate in Golgi transport were analyzed. Only proteins with validated protein–protein binding were included in the analysis. The tool “Path Explorer” in the IPA software was used to generate the interactome. Gray lines are interactions found by the software in IPA databases. Blue lines indicate known interaction validated from current literature by IP, co-IP, yeast two-hybrid screen, tubulin-binding assay, and affinity column purification. Species were limited to human, mouse, and rat.

The software failed to find interacting partners for four proteins (Figure 5), the leucine rich repeats and guanylate kinase domain containing (LRGUK), zona pellucida-binding protein 1 (ZPBP1), GALNT3 and AU040320. LRGUK has been recently shown to be essential for sperm head shaping, via the manchette. *Lrguk1*-mutant mice have abnormal sperm head shaping, detached acrosomes, and the absence of axoneme extension from the basal body [134]. On the other hand, ZPBP and GALNT3 have been shown to be important for acrosome biogenesis and sperm morphology [125,144]. Finally, AU40320-knockout mice are infertile and present a globozoospermia-like phenotype. Spermatozoa are immotile and display a malformed roundish head with no acrosome. In round spermatids, proacrosomal vesicles accumulate close to the acroplaxome but fail to fuse into a single acrosomal vesicle. These results suggest that AU040320 may be necessary for the normal formation of proacrosomal vesicles or the recruitment of cargo proteins required for downstream events leading to acrosomal fusion [39]. 

Interestingly, 23 new proteins were identified in the interactome by the software. Among them, APC, UBE2, HSP90, and VCP appeared as in IMT. Aquaporin 1 (AQP1) is a member of the AQP family. Aquaporins play a vital role in the transport of water and solutes across cell membranes [149]. TNF receptor-associated factor 2 (TRAF2) is known to regulate NFκB signaling in germ cells [164]. Polycystin 2 (PKD2) protein is an integral transmembrane protein with non-selective cation channel activity which has been suggested to play a role in the acrosome reaction [162]. Histone demethylase KDM1A (lysine-specific demethylase 1A) is important for the differentiation and maintenance of spermatogonia in mice [156]. G Protein Subunit Beta 5 (GNB5) is a heterotrimeric guanine nucleotide-binding protein that integrates signals between receptors and effector proteins. Solute Carrier Family 25 Member 6 (SLC25A6), also known as ANT3, is a protein that catalyzes the exchange of cytoplasmic ADP with mitochondrial ATP across the mitochondrial inner membrane [152]. DDX6 (DEAD [Asp-Glu-Ala-Asp] box helicase 6 is a member of the ATP-dependent DEAD-box RNA helicase family, which is abundantly expressed in several tissues. DDX6 has been shown to localize to the nucleus and in outer dense fibers [150]. Pyruvate Dehydrogenase E1 Subunit Alpha 1 (PDHA1) has been shown to be expressed during spermatogenesis [161].

A number of phosphatases and kinases were also found to connect with this interactome, including the phosphoprotein phosphatase 1 catalytic subunit alpha (PPP1CA) [163], AKT1 [147], Glycogen Synthase Kinase 3 Beta (GSK3B) [153], NEK4 [160], guanylate kinase 1 (GUK1) [154] and Leucine Rich Repeat Kinase 2 (LRRK2) [157]. Moreover, motor proteins, including dynein cytoplasmic 1 heavy chain 1 (DYNCH1) and the kinesins proteins KIF5C and KIFC3, are also part of the interactome [151]. Additional proteins found are enzymes associated with the proteasome machinery, including Mindbomb E3 Ubiquitin Protein Ligase 1 (MIB1) [158] and Mitochondrial E3 Ubiquitin Protein Ligase 1 (MUL1) [159].

New partners were found for some proteins that are present in both IMT and Golgi transport. NEK4 appears in this interactome as a new partner for SPAG17 as well as SPINK2 for SPAG6. APC also shows interaction with two new proteins, HRB and GSK3B. FU has six new connections, LRRK2, GSK3B, PPP1CA, AKT1, GM130 and TRAF2. VPC has four new connections, IFT74, GOLGA3, RNF19A and MUL1. GOPS5 has six new interactions—DYNC1H1, HSP90B1, HSP90, SLC25A6, TRAF2, and GNB5. Further studies may elucidate the functional significance of these complexes in Golgi transport and acrosome biogenesis.

Interestingly, GOPS5 does not interact with HSP90 in IMT, but they interact in Golgi transport. Conversely, IFT88 and IFT20 interact in IMT, but are no longer interacting partners in Golgi transport. It is unclear why these two IFT are no longer connected in the Golgi transport. IFT88 has been shown to be expressed during early spermiogenesis and to participate in the development of the acrosome–acroplaxome complex, as well as in the development of the flagellum [52]. However, germline-specific loss of IFT20 causes male infertility, but whether acrosome biogenesis is affected in these mice has not been determined [30]. 

### 4.4. Novel Technology to Study Trafficking of Proteins (Super-Resolution Microscopy) 

Cell morphology has been classically probed by optical microscopy and transmission electron microscopy (TEM). Conventional optical microscopy is fast and inexpensive, but it is limited in resolution by a fundamental property of light waves, namely the diffraction limit. As light is transmitted through an objective, it is diffracted by the lens aperture, introducing a minimum size for the image of a point source. As a consequence, images are blurred by this diffraction effect and, for practical purposes, the resolution cannot surpass 200 nm. On the other hand, the diffraction limit for electron waves in transmission electron microscopes is typically three orders of magnitude smaller than that of optical microscopy. For a 100 kV TEM, the diffraction limit is only of the order of 0.22 nm and, therefore, in these systems, the resolution is usually defined by the distortions introduced by different elements in the microscope. Extremely high-resolution images are possible in electron microscopes. TEM, however, is labor-intensive, measurements are usually invasive, and they lack the specificity that characterizes fluorescence microscopy, where a fluorophore is bound to the molecule of choice. 

In order to take advantage of the non-invasive and highly specific nature of fluorescence microscopy, a number of new techniques have been developed to surpass the diffraction limit [216]. We collectively refer to these imaging tools as super-resolution microscopy techniques. While a myriad of acronyms has emerged to describe different super-resolution techniques, the typical ones can be classified as structured illumination, single-molecule localization, and stimulated-emission depletion (STED) [217]. Structured illumination microscopy (SIM) bypasses the diffraction barrier by employing a non-traditional illumination pattern with intensities that vary sinusoidally in space. Single-molecule localization microscopies are known as photoactivatable localization microscopy (PALM) or stochastically optical reconstruction microscopy (STORM). PALM uses photoactivatable proteins and STORM employs fluorophores that blink. These methods achieve super-resolution by detecting a small random subset of molecules at any given imaging frame. Thus, the position of each individual fluorophore can be obtained with very high precision. By collecting many frames, a high-resolution image is reconstructed from all the fluorophore localizations. STED accomplishes super-resolution by introducing a second beam, usually with a donut shape, that forces molecules to become dark in this region. This beam is tuned to have a wavelength that depletes fluorophores via stimulated emission. Consequently, only the molecules in the center of the donut (i.e., in the donut hole) can emit fluorescence light. The impact in the life sciences of these new imaging modalities cannot be overemphasized and it has led to the 2014 Nobel Prize in Chemistry. Single-molecule localization microscopy, in particular, allows not only to image the localization of specific molecules or complexes within a cell, but it also enables following the path of a molecule in a live cell in real time [218,219]. 

**Figure 6:**
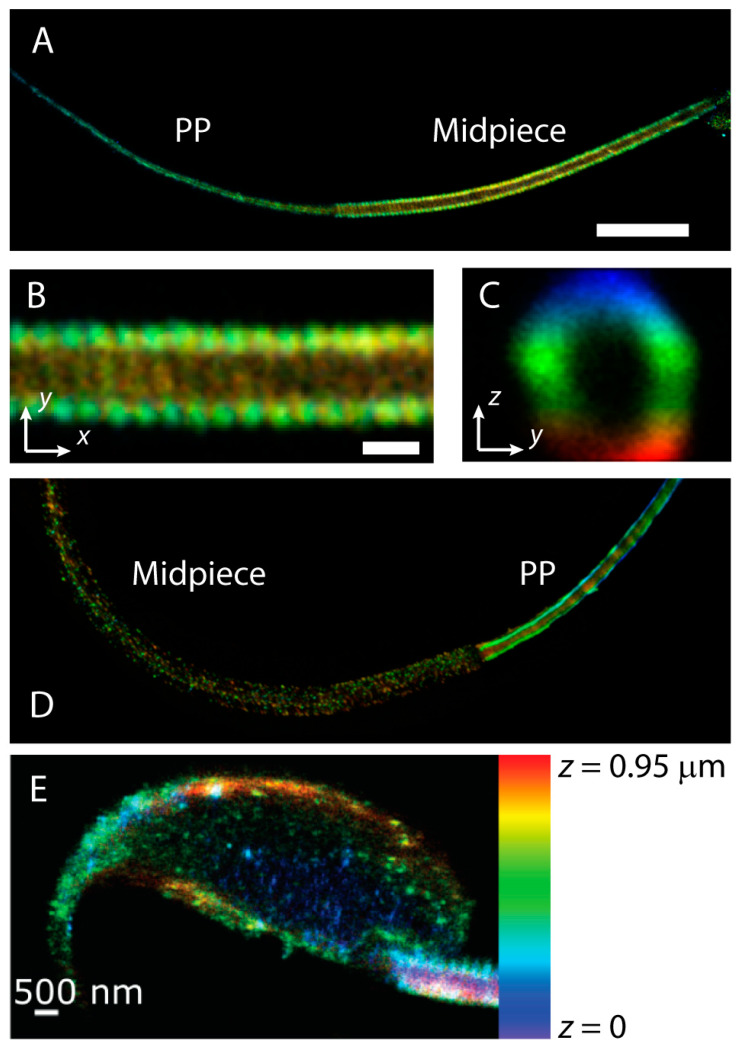
Examples of stochastically optical reconstruction microscopy (STORM) super-resolution imaging in mouse sperm cells. (**A**) Representative color-coded 3D STORM reconstruction of the actin cytoskeleton in the flagellum of mouse sperm. Actin was labeled with phalloidin-AlexaFluor 647. The midpiece and the principal piece (PP) are indicated for clarity. Scale bar: 5 μm. Imaged by Xinran Xu and Maria G. Gervasi [223]. The actin structure in the midpiece is found to be arranged in a double helix. (**B**) Zoom of the midpiece structure. Scale bar: 500 nm. (**C**) Cross section of the midpiece structure. (**D**) Representative 3D STORM reconstruction of actin-associated protein adducin in the mouse sperm flagellum. (**E**) Representative 3D STORM reconstruction of the actin cytoskeleton in the head of non-capacitated mouse sperm. Imaged by Xinran Xu and Mariano Buffone [228]. The color bar on the right shows the axial localization of this reconstruction.

Imaging the main events in spermiogenesis, such as the formation of the flagellum and the acrosome, the condensation of chromatin in the nucleus, and the removal of unnecessary cytoplasm and organelles, using super-resolution microscopy could provide valuable information. These tools have already proved instrumental in answering difficult cell biology questions in different systems. To cite a few examples, super-resolution microscopy enabled for the first time the visualization of diverse actin-based structures such as periodic rings in neuronal axons [220,221], a fractal in the vicinity of the plasma membrane of human embryonic kidney cells [222], and a double helix in the midpiece of murine sperm (Figure 6A–D) [223]. Super-resolution studies have also aided in unraveling the molecular organization in neuronal synapses [224] and within plasma membrane nanodomains [225,226,227]. 

Within the realm of germ cells, spermatozoa have already been established as an excellent system for investigations employing super-resolution imaging. In these cells, the best-known success of super-resolution imaging lies in the discovery of the spatial organization of the sperm-specific calcium channel CatSper [229,230]. CatSper is a key player in the regulation of Ca^2+^ influx in the sperm flagellum and loss of function mutations cause male infertility. Ca^2+^ influx triggers sperm hyperactivation, a powerful asymmetric motion of the tail characteristic of capacitated sperm, that is necessary for sperm migration in the oviduct and penetration of the zona pellucida surrounding the oocyte. Using 3D STORM, Chung and co-workers observed that CatSper forms a unique pattern of four linear domains in the plasma membrane of the flagellar principal piece [229]. Interestingly, the authors of this report found that the Ca^2+^ signaling molecules caveolin1, CaMKII, and calcineurin localize to the CatSper quadrilateral domains and that the genetic deletion of CatSper produces the delocalization of these signaling proteins. Later, the same group reported that the deletion of the subunit CatSperζ disrupts the continuity of the CatSper linear domains. A result that was verified using both 3D STORM and STED [230]. Recently, using an imaging strategy known as 4Pi single-marker switching nanoscopy (4Pi-SMSN), it was found that each CatSper quadrant is actually formed by two rows and that the auxiliary subunit EFCAB9 controls this substructure by binding to CatSperζ in a Ca^2+−^dependent manner [231]. The 4Pi-SMSN employs two objectives to generate an interference pattern at the detector, which can improve 7-fold the localization accuracy in the axial direction.

Hyperactivation is a physical manifestation of capacitation, a process that takes place in the female reproductive tract by which sperm acquire the capacity to fertilize and are prepared to undergo acrosomal exocytosis. The cellular changes associated with the capacitation process are highly complex and include an increase in intracellular Ca^2+^, cyclic AMP (cAMP) synthesis by soluble adenylyl cyclase, intracellular alkalinization, hyperpolarization of the plasma membrane potential, and extensive tyrosine phosphorylation [232]. Super-resolution imaging has been used to study the localization of signaling molecules within the sperm compartments and their redistribution during the capacitation process. These measurements have been shown to be consequential in the understanding of spatio-temporal regulation of capacitation. The 3D STORM has been employed to accurately dissect the distribution of FER, the protein responsible for the capacitation-associated increase in tyrosine phosphorylation [233]. FER localization overlaps with the localization of tyrosine phosphorylation in both midpiece and principal piece. A prominent aspect of capacitation involves phosphorylation signaling cascades that are initiated by protein kinase A (PKA). This protein is a broad-spectrum Ser/Thr kinase and, thus, its activation must be precisely regulated in time at specific locations. The redistribution of PKA during capacitation was directly revealed by 3D STORM [232]. 

In addition to capacitation-associated signaling pathways in the sperm flagellum, super-resolution has been employed to investigate the acrosome reaction. Namely, it has revealed changes in the actin cytoskeleton in the sperm head (Figure 6E) [228], the distribution of specific integrin heterodimers [234,235], and the formation of SNARE protein complexes immediately prior to acrosome exocytosis [236]. 3D STORM has also revealed unique structures in sperm cells. It was found that the voltage-gated proton channel Hv1 forms two asymmetric lines that run along the flagellum of human sperm [237]. This work suggests the positioning of Hv1 domains plays important roles in sperm motility. Further, specialized actin structures were discovered in the sperm midpiece and principal piece (Figure 6A–D) [223]. Strikingly, in the midpiece of mouse sperm, polymerized actin forms a double helix that follows the mitochondrial sheath, a type of filamentous actin structure that had not been previously observed.

The use of super-resolution as a tool in spermiogenesis research is only in its initial stages. The most popular super-resolution modality that has been used to study spermatids is based on SIM. While this tool cannot reach the tens of nanometers in resolution that is possible with single-molecule localization microscopy or STED, it can still double the resolution achievable by conventional microscopy. SIM was employed to image membrane-associated RING-CH 10 (MARCH10) proteins, which are expressed in elongated and elongating spermatids but are not expressed in sperm cells. Confocal microscopy reveals that MARCH10 localizes to the principal piece of elongating spermatids, suggesting it plays a role in the formation of the sperm flagellum [238]. Further, 3D SIM super-resolution microscopy in spermatids reveals MARCH10 forms two dotted strands parallel to each other in the principal piece. It has also been found to form a ring-like structure in the annulus [238], a septin-based structure that acts as a diffusion barrier between the midpiece and the principal piece [239,240]. Protein kinase A-anchoring protein 4 (AKAP4) also appears with a similar two-strand structure in 3D SIM images of spermatid flagella, but its localization is closer to the plasma membrane than that of MARCH10 [238]. 

The 3D-SIM revealed that two classes of centrosomin proteins, CnnT and CnnT-C, reside on the surface of mitochondria in *Drosophila* spermatids [241]. CnnT was shown to recruit and activate the g-tubulin ring complex, generating microtubule-organizing centers on the mitochondrial surface to support spermiogenesis. In addition, the ciliary transition zone has been studied in both spermatogonia and spermatids of *Drosophila* [242]. Using super-resolution imaging, this study has shed light on the growth of axoneme microtubules in spermatocytes and the elongation of sperm flagella. 

There are very few examples where super-resolution microscopy beyond SIM has been used to image male germ cells. STED was exploited to visualize the acrosomal membrane [243]. For this purpose, spermatids were obtained from a transgenic mouse expressing fluorescent equatorin, a fertilization-related protein that localizes to the outer acrosomal membrane of the principal and equatorial segment and at the inner acrosomal membrane. This study demonstrated the capability of STED microscopy to distinguish the different membranes in the sperm head, identifying super-resolution microscopy as a powerful tool in the investigation of the players involved in the formation of the acrosome and in the acrosome reaction.

## 5. Conclusions and Future Directions

This review integrates recent findings regarding protein trafficking during sperm differentiation. The complete interactome of the proteins involved in Golgi transport and IMT mechanisms remains to be characterized. Here, we performed an in silico protein interaction analysis as a tool to generate models for the interactome based on the current literature. Future investigations should be performed to validate these two models. 

As mentioned previously, super-resolution techniques are cutting edge technology that allow investigators to study protein trafficking. Several advances have been made using this technology, and it is expected that, in the near future, new algorithms will provide further improvement of the resolution of the images. Another technology that provides high resolution at the molecular level is electron cryotomography (Cryo-ET). The resolution of this technology reaches 1–4 nm, and the combination of series of 2D images generates a 3D view of the samples. 

Several issues remain unresolved regarding protein trafficking in the manchette. For example, there is controversy about the nucleation of microtubules that originate the manchette assembly. Moreover, post-translational modifications of tubulin and actin filament tracks have not been fully characterized. Future studies should help elucidate the spatial and temporal coordination of the protein trafficking mechanisms, and how these processes regulate the differentiation of spermatids to spermatozoa.

Understanding the interactome and the protein trafficking mechanisms during sperm differentiation has clinical implications. Defects in sperm differentiation lead to male infertility, congenital disorders, and spontaneous abortion. Therefore, it is important to focalize our scientific efforts on developing new strategies to dissect the interactome of proteins and the mechanisms of protein transport in spermiogenesis. 

## Figures and Tables

**Table 1:** Actin-related proteins playing a role during sperm differentiation.

Protein	Function	Specie	References
α-ACTININ	Structure and stabilization of the cytoskeleton	Bovine	[59]
ARC	Possible role in the acrosome formation and the sperm acrosome reaction	Mouse	[60]
ARPM1	Germ cell morphogenesis	Mouse	[61]
ARP2/3	Actin polymerization	Mouse, rat, guinea pig	[45]
ARP3	Actin nucleation and branching	Rat	[62]
CALICIN	Stabilization of the cytoskeleton	Human, mouse, rat, boar, guinea pig, bull	[63]
CAPZA3	Maintains polymerized actin during spermiogenesis	Mouse	[64,65]
CDC42	Actin reorganization	Rat	[66]
CORTACTIN	Arp2/3 complex activation; formation of actin network	Rat	[62]
LIMK2	Cofilin inactivation; germ cell development	Mouse	[67]
MDIA1/2	Actin nucleation	Rat	[68]
MYOSIN	Molecular motors	Human, mouse, rat, bovine	[69]
N-WASP	Arp2/3 complex activation	Rat	[62]
PROFILIN III	Actin monomer binding; germ cell morphogenesis	Mouse	[61]
PROFILIN IV	Actin monomer binding; germ cell morphogenesis	Human, mouse, rat	[70]
RAC1	Actin reorganization	Rat	[66]
RHOB	Actin reorganization; regulation of Sertoli–germ cell adhesion	Rat	[71]
T-ACTIN 1/2	Germ cell morphogenesis/sperm function	Mouse	[72]
TESTIS FASCIN	Actin monomer binding; germ cell morphogenesis	Human, mouse	[73]
WAVE1	Arp2/3 complex activation; germ cell morphogenesis/sperm function	Human, mouse, bull, baboon	[74]

**Table 2:** Kinesins playing a role during sperm differentiation.

Stages	Kinesin Type	Functions	Species	Reference
Acrosome biogenesis	KIFC1	Transport vehicles; tether acrosome to the nucleus	Rat and crustaceans	[77,78]
	KRP3A, KRP3B	Transport vehicles like Golgi	Rat and bull	[79]
	KIF5C	Redistribution of proteins related to acrosome formation	Mouse	[80]
Nuclear shaping	KIFC5A	Interact with manchette and promote nuclear shaping	Mouse	[81]
	KIFC1	Link the nucleus to the manchette; promote nucleus condensation and elongation	Mouse and *Octopus tankahkeei*	[82,83]
	KIF17B	Promote nuclear shaping in a manchette-dependent way	Mouse	[84]
	KIF3A	Manchette organization and sperm head shaping	Mouse	[85]
Tail formation	KIF17B	Intraflagellar transport	Rat	[86]
	KIF3A	Axoneme formation	Mouse	[85]
	KLC3	Transport mitochondria to midpiece	Rat	[87]
Spermatid maturation	KIF20	Spermatid translocation	Rat and mouse	[88]
	KRP3	Spermatid translocation	Rat	[89]
Spermatid transcription	KIF17B	ACT subcellular distribution	Monkey	[90]
	KIF17B	Transport CREM mRNA	Mouse	[91]
	KIF17B	Chromatoid body movement, RNA metabolism	Mouse	[92]

**Table 3:** List of proteins known to localize to the Golgi, Golgi vesicles, acrosome and/or acroplaxome, whose interacting partners were confirmed by immunoprecipitation (IP), co-immunoprecipitation (co-IP), yeast two-hybrid (Y2H) screen and/or IPA database (affinity chromatography, co-IP, anti-bait co-IP, enzyme activity assay, in situ proximity ligation assay, IP, liquid chromatography mass spectrometry, pulldown assay, tandem affinity purification, Western blotting, and yeast two-hybrid screen). Species were limited to human, mouse, and rat.

Protein	Type of Interaction Confirmation	Reference
ACTIN	IPA	[119]
ARP2/3	IPA	[45]
ATG7	IPA	[41]
AU040320	―	[39]
CEP131	IPA	[120]
CCDC181	IPA	[121]
CLATHRIN	IPA	[103]
COPS5	Co-IP, Y2H, and IPA	[122]
CORTACTIN	IPA	[47]
CSNK2A2	IPA	[80]
DYP19L2	IPA	[123]
FERT	IPA	[47]
FU	IPA	[124]
GALNT3	IPA	[125]
GCNF	IPA	[126]
GOLGA3	IPA	[113,114,115]
GMAP210	IPA	[28,127]
GM130	IPA	[128]
GOPC	IPA	[129]
HRB	IPA	[130]
HSP90B1	IPA	[131]
IFT20	Co-IP, Y2H, and IPA	[30,132]
IFT74	IPA	[33]
IFT88	IPA	[28]
KATNB1	IPA	[133]
LRGUK1	―	[134]
MGCRABGAP	Co-IP	[99,135]
MNS1	IPA	[85]
MORN3	IPA	[136]
MYOSIN	IPA	[44]
PICK1	IPA	[111]
PSMC3	IPA	[137]
RAB3A	Co-IP	[135]
RAB27	IPA	[138]
RIMBP3	IPA	[139]
RNF19A	IPA	[137]
SIRT1	IPA	[129]
SMAP2	IPA	[109,112]
SNAPIN	Y2H	[26]
SPACA1	IPA	[140,141]
SPAG6	Co-IP, Y2H, and IPA	[26]
SPAG16	Co-IP and IPA	[142]
SPAG17	IPA	[21]
SPEF2	Co-IP, Y2H, and IPA	[132]
SPINK2	Y2H	[26]
STK36	IPA	[124]
TMF	IPA	[143]
α/β-TUBULIN	IPA	[144]
VPS54	IPA	[145]
ZPBP	―	[146]
**New Proteins Detected by IPA Software**		
AKT1	IPA	[147]
APC	IPA	[148]
AQP1	IPA	[149]
DDX6	IPA	[150]
DYNC1H1	IPA	[151]
GNB5	IPA	[152]
GSK3B	IPA	[153]
GUK1	IPA	[154]
HSP90	IPA	[155]
KDM1A	IPA	[156]
KIF5C	IPA	[151]
KIFC3	IPA	[151]
LRRK2	IPA	[157]
MIB1	IPA	[158]
MUL1	IPA	[159]
NEK4	IPA	[160]
PDHA1	IPA	[161]
PKD2	IPA	[162]
PPP1CA	IPA	[163]
SLC25A6	IPA	[152]
TRAF2	IPA	[164]
UBE2	IPA	[165]
VCP	IPA	[166]

**Table 4:** List of proteins known to localize to the manchette and their interacting partners confirmed by immunoprecipitation (IP), co-immunoprecipitation (co-IP), yeast two-hybrid (Y2H) screen, microtubule binding assay (MBA), tubulin-binding assay (TBA) and/or IPA database (affinity chromatography, co-IP, anti-bait co-IP, enzyme activity assay, in situ proximity ligation assay, IP, liquid chromatography mass spectrometry, pulldown assay, tandem affinity purification, Western blotting, and yeast two-hybrid screen). Species were limited to human, mouse, and rat.

Protein	Type of Interaction Confirmation	Reference
ACTIN	Co-IP, IP, Y2H, and IPA	[178]
ARP1	IPA	[179]
CALMODULIN	IPA	[180]
CBE1	―	[181]
CCDC42	Co-IP and IPA	[176,177]
CCDC181	Y2H	[121]
CDC42	IPA	[182]
CLIP-170	IPA	[183]
CNTROB	Co-IP	[184]
COPS5	Co-IP, Y2H, and IPA	[122]
DYNEIN	IPA	[98,179]
FAM46C	―	[185]
FU	IP and IPA	[124]
GCNF	―	[126]
GMAP210	IPA	[127]
GSTO2	IPA	[186]
HOOK1	Co-IP, IP, MBA, Y2H, and IPA	[121,134,139,187]
HOOK2	Co-IP, IP, MBA, Y2H, and IPA	[134,187,188]
HOOK3	Co-IP, IP, MBA, Y2H, and IPA	[187,188]
IFT20	Co-IP, IP, Y2H, and IPA	[30,122,127,132,189]
IFT27	IPA	[32]
IFT88	IPA	[28]
IFT172	IPA	[35]
IQCG	IPA	[180,190]
KATNAL2	Co-IP and IPA	[191]
KATNB1	IPA	[192]
KIF3A	Co-IP, MBA, and IPA	[85]
KIF3B	Y2H and IPA	[85,139]
KIF17B	Co-IP and IPA	[84]
KIF5C	IPA	[81]
KIF27	IP	[124]
KLC3	Co-IP, IP, Y2H, and IPA	[188]
KRT5	Co-IP and IPA	[28,184]
LIS1	TBA and IPA	[193]
LRGUK1	Co-IP, IP, and Y2H	[134,188]
MGCRABGAP	Co-IP	[99,135]
MEIG1	Co-IP, Y2H, and IPA	[194,195]
MNS1	Co-IP	[85]
MORN3	Co-IP and Y2H	[136]
MYRIP	IPA	[98]
MYOSIN	IPA	[44,98]
NESPRIN1	IPA	[50]
ODF1	Co-IP, IP, and IPA	[124]
PACRG	IPA	[194,195]
PFN3	IP and Y2H	[48]
PFN4	―	[48]
PSMC3	Co-IP	[137]
RAB3A	Co-IP and IPA	[135]
RAB10	Co-IP and IPA	[99]
RANBP17	Co-IP and Y2H	[175]
RAB27	IPA	[98]
RIMBP3	Co-IP and Y2H	[139,188]
RNF19A	Co-IP	[137]
SEPT12	Co-IP and IPA	[182,196,197,198]
SNAPIN	Y2H and IPA	[26]
SPAG6	Y2H and IPA	[20,22,25,26]
SPAG16	Co-IP and IPA	[142,194]
SPAG17	IPA	[21]
SPATIAL	Co-IP	[84]
SPEF2	Co-IP, Y2H, and IPA	[132,199]
SPEM1	Co-IP and Y2H	[91,92,120,135,136]
STK33	Co-IP and IP	[200]
STK36	IPA	[124]
SUN3	IPA	[50]
SUN4	IPA	[197,201,202]
TMCO5A	―	[203,204]
α/β-TUBULIN	IPA	[178]
δ-TUBULIN	Co-IP and IPA	[191]
ε-TUBULIN	Co-IP and IPA	[191]
γ-TUBULIN	Co-IP and IPA	[191]
UBQLN1	Co-IP and Y2H	[174]
WBP2	IPA	[205]
**New Proteins Detected by IPA Software**		
APC	IPA	[148]
BSG	IPA	[206]
EGFR	IPA	[207]
GPX4	IPA	[208]
GSK3B	IPA	[209]
HSP70	IPA	[210]
HSP90	IPA	[155]
KATNA1	IPA	[133]
LAMINB1	Co-IP	[197]
MAP3K11	IPA	[211]
UBE2	IPA	[165]
VCP	IPA	[166]
